# Protein tyrosine kinase 6 is associated with nasopharyngeal carcinoma poor prognosis and metastasis

**DOI:** 10.1186/1479-5876-11-140

**Published:** 2013-06-09

**Authors:** Li-na Liu, Pei-yu Huang, Zhi-rui Lin, Li-juan Hu, Jian-zhong Liang, Man-zhi Li, Lin-quan Tang, Mu-sheng Zeng, Qian Zhong, Bo-hang Zeng

**Affiliations:** 1 Department of Oncology, the Second Affiliated Hospital of Guangzhou medical college, 250 Changgang Road East, Guangzhou 510260, China; 2 State Key Laboratory of Oncology in South China, Sun Yat-sen University Cancer Center, Guangzhou, China; 3 Department of Nasopharyngeal Carcinoma, Sun Yat-sen University Cancer Center, Guangzhou, China; 4 Department of Medical Oncology, Affiliated Tumor Hospital of Guangzhou Medical College, 651 Dongfeng Road East, Guangzhou 510060, China; 5 Departmemt of Pathology, Sun Yat-sen University Cancer Center, Guangzhou, China

**Keywords:** PTK6, Nasopharyngeal carcinoma (NPC), Prognostic biomarker, Immunohistochemistry

## Abstract

**Background:**

The aim of this study was to analyze the expression of protein tyrosine kinase 6 (PTK6) in nasopharyngeal carcinoma (NPC) samples, and to identify whether PTK6 can serve as a biomarker for the diagnosis and prognosis of NPC.

**Methods:**

We used quantitative RT-PCR and Western blotting analysis to detect mRNA and protein expression of PTK6 in NPC cell lines and immortalized nasopharyngeal epithelial cell lines. 31 NPC and 16 non-tumorous nasopharyngeal mucosa biopsies were collected to detect the difference in the expression of mRNA level of PTK6 by quantitative RT-PCR. We also collected 178 NPC and 10 normal nasopharyngeal epithelial cases with clinical follow-up data to investigate the expression of PTK6 by immunohistochemistry staining (IHC). PTK6 overexpression on cell growth and colony formation ability were measured by the method of cell proliferation assay and colony formation assay.

**Results:**

The expression of PTK6 was higher in most of NPC cell lines at both mRNA and protein levels than in immortalized nasopharyngeal epithelial cell lines (NPECs) induced by Bmi-1 (Bmi-1/NPEC1, and Bmi-1/NPEC2). The mRNA level of PTK6 was high in NPC biopsies compared to non-tumorous nasopharyngeal mucosa biopsies. IHC results showed the expression of PTK6 was significantly correlated to tumor size (P<0.001), clinical stage (P<0.001), and metastasis (P=0.016). The patients with high-expression of PTK6 had a significantly poor prognosis compared to those of low-expression (47.8% versus 80.0%, P<0.001), especially in the patients at the advanced stages (42.2% versus 79.1%, P<0.001). Multivariate analysis indicated that the level of PTK6 expression was an independent prognostic factor for the overall survival of patients with NPC (P <0.001). Overexpression of PTK6 in HNE1 cells enhanced the ability of cell proliferation and colony formation.

**Conclusions:**

Our results suggest that high-expression of PTK6 is an independent factor for NPC patients and it might serve as a potential prognostic biomarker for patients with NPC.

## Background

Nasopharyngeal carcinoma (NPC) is not a common tumor in most parts of the world, but there is a high incidence in certain areas of southern China, South-Asia and North Africa [1,2]. Epstein-Barr virus (EBV) infection, genetic susceptibility and environmental carcinogens are all believed to be the etiologic factors associated with NPC [3]. NPC is different from other head and neck malignancies, because of its highly invasive and metastatic features [4]. Most patients can be cured when they were diagnosed and treated at early stages since primary NPC is sensitive to radiotherapy. However, at the advanced stages, distant metastasis is the main cause of treatment failure [5,6].

By now, clinical TNM (Tumor, Node, Metastasis) staging is often used to determine the prognosis of NPC patients. However, the NPC patients at the same clinical stage usually have different clinical outcomes. It is suggested that TNM staging is deficient in predicting the prognosis of NPC accurately [7,8]. With the development of biomedical research, some molecular biomarkers have been identified to be associated with NPC prognosis, such as plasma EBV DNA, serum amyloid protein A (SAA), sailic acid (SA), EGFR and CENP-H. [9-14].

Protein tyrosine kinase 6 (PTK6), also known as the breast tumor kinase (BRK), is an intracellularly non-receptor Src-related tyrosine kinase [15,16]. It was initially cloned from a metastatic breast tumor [17]. The PTK6 gene is located in chromosome 20q 13.3-13.4 and consists of 8 exons. It is composed of Src homology 2 (SH2) domain, Src homology 3 (SH3) domain, and protein kinase domain. Since PTK6 lacks myristoylation and palmitoylation signals, the subcellular localization becomes greater flexibility. The expression of PTK6 has been found in normal differentiated epithelial cells including esophagus [18], prostate [19], gastrointestinal tract [20,21], skin [22,23], oral epithelium [24], and lymphocytes [25]. It is reported that PTK6 can negatively regulate epithelial cell growth and induce apoptosis in normal intestine [26,27]. It has also been reported that PTK6 can induce apoptosis of the epithelial cells of the intestine and skin by DNA damage and serum derivation [28]. Stephanie Ma et al. found that PTK6 may be an important tumor suppressor in esophageal squamous cell carcinoma development. They also found the downregulation of PTK6 could promote tumorigenicity and metastasis [18]. However, over-expression of PTK6 is a common phenomenon in a variety of epithelial tumors, such as breast cancer [15,29,30], ovarian cancer [31], colon cancer [20], head and neck cancer [32], non-small cell lung cancer (NSCLC) [33], and metastatic melanoma [34]. For instance, the elevated expression of PTK6 was detected in breast cancer cell lines and more than 65% primary breast cancers, but was undetectable or at a low level in normal mammary tissue and benign lesions [29,30]. Over-expression of PTK6 was shown to increase proliferation, anchorage-independent growth, cell migration, and tumor growth in many kinds of breast cancer model system, while knocking-down of PTK6 leaded to the opposite results [15]. Also, PTK6 gene was amplified at low levels in primary ovarian cancer and its protein level was highly expressed in the majority of high-grade serous carcinomas and ovarian cancer cell lines but not the normal ovary [31]. Moreover, PTK6 high mRNA expression has been reported in the cancers of the bladder [35], pancreas [36], and gastric cancer [37], but its protein expression and functions have not been validated in these cancers. Many studies have shown that PTK6 play different roles in normal and cancer epithelia, suggesting that the function of PTK6 may associate with its intracellular localization (cytosol or nuclei) [15,38,39]. Nevertheless, the role of PTK6 in the tumorigenesis of NPC has not been explored, to date.

In this study, our aim was to detect the expression of PTK6 in NPC, and identify whether PTK6 can be a potential diagnostic and prognostic biomarker for NPC patients. To examine PTK6 mRNA and protein expression, quantitative RT-PCR, Western blotting analysis and immunohistochemistry (IHC) methods were applied. Then we analyzed the relationship of PTK6 expression and the clinical factors as well as the prognosis of NPC patients. In addition, overexpression PTK6 can enhance the proliferation and colony formation ability in NPC cells. Finally, our findings indicate that high PTK6 expression may play an important role in NPC progression and serve as an independent prognostic biomarker for forecasting poor prognosis in NPC patients, peculiarly those with advanced clinical stages.

## Materials and methods

### Nasopharyngeal patients and clinical tissue specimens

All clinical samples used for the expression of PTK6 studies by quantitative RT-PCR, and immunohistochemistry (IHC) assay were collected from Sun Yat-sen University Cancer Center (SYSUCC), Guangzhou, China. 31 NPC and 16 non-tumorous nasopharyngeal mucosa biopsies were collected for quantitative RT-PCR assay during 2011. Each biopsy specimen was immersed into the RNA-Later reagent overnight at 4°C and then preserved at −80°C prior to RNA extraction. For IHC analysis, 178 paraffin-embedded NPC specimens and 10 normal nasopharyngeal epithelial samples were collected between 2005 and 2007. The clinicopathological characteristics are summarized in Table 1. All of these NPC patients were treated with standard curative radiotherapy with or without chemotherapy. Cancer TNM stage was defined on the basis of the UICC (International Union Against Cancer) and the AJCC (American Joint Committee on Cancer). All the patients were followed from the date of diagnosis until death or the lasted census date. The Institute Research Medical Ethics Committee of Sun Yat-Sen University granted approval for this study.

**Table 1:** Characteristics of 178 NPC patients

**Characteristic**	**Nasopharyngeal carcinoma patients (N=178) %**
**Sex**	
Female	45 (25.3)
Male	133 (74.7)
**Age (years)**	
Median (range)	46 (19–75)
≤46	99 (55.6)
≤46	79 (44.4)
**Follow-up time (months)**	
Median (range)	56 (3–89)
**Tumor size**	
T1+T2	58 (32.6)
T3+T4	120 (67.4)
**Lymphoid nodal states**	
N0-1	92 (51.7)
N2-3	86 (48.3)
**Clinical stage**	
I+II	33 (18.5)
III+IV	145 (81.5)
**Local-regional relapse**	
Yes	23 (12.9)
No	155 (87.1)
**Metastasis**	
Yes	33 (18.5)
No	145 (81.5)
**WHO histological classification**	
NKUC	170 (95.5)
NKDC	8 (4.5)
**OS rate (%)**	
5-year	53.9

Abbreviations *NPC* nasopharyngeal carcinoma, *WHO* World Health Organization, *NKUC* non-keratinizing undifferentiated carcinoma, *NKDC* non-keratinizing differentiated carcinoma, *OS* overall survival.

### Cell lines and cell cultures

Two immortalized nasopharyngeal epithelial cell lines (NPECs) induced by Bmi-1 (NPEC1 Bmi-1 and NPEC2 Bmi-1) were established as described previously and cultured in keratinocyte serum-free medium (KSF, Invitrogen, USA) [40,41]. The NPC cell lines including CNE1, CNE2, 6-10B, 5-8F, HONE1 , SUNE1 and HNE1 were cultured in RPMI-1640 medium (Gibco, USA) with 5% fetal bovine serum (Gibco, USA). All cell lines were grown in a humidified incubator 37°C with 5% CO_2_.

### RNA extraction and quantitative RT-PCR analysis

Total RNA of NPC and non-tumorous nasopharyngeal mucosa biopsies were extracted from the E.Z.N.A. total DNA/RNA isolation kit according to manufacturer’s instructions (OMEGA bio-tek; R6731; USA). The RNA from various cell lines was extracted as described previously [42]. RNA concentration and quantity were determined with NanoDrop spectrophotometer (ND-1000, Thermo Scientific, USA). According to the manufacturer’s instructions, the first strand cDNA synthesis was performed using 1 μg of total RNA and M-MLV reverse transcriptase (Invitrogen, USA). We used iQ™ SYBR Green Supermix to determine the threshold cycle (Ct) value of each specimen in the CFX96 real-time PCR detection system (Bio-Rad, CA, USA). Glyceraldehyde-3-phosphate dehydrogenase (GAPDH) was used as an internal control for comparison and normalization in these studies.

The following primers were used:

PTK6 forward 5^′^-TACTTTGGGGAGGTCTTCGAG-3^′^;

PTK6 reverse 5^′^-TGCCGCAGCTTCTTCATG-3^′^;

GAPDH forward 5^′^- CTCCTCCTGTTCGACAGTCAGC-3^′^;

GAPDH reverse 5^′^-CCCAATACGACCAAATCCGTT-3^′^.

Quantitative RT-PCR amplifications were performed under this condition: UDG incubation at 50°C for 2 min, initial denaturation at 95°C for 2 min, denature at 95°C for 15 s, anneal and extend at 60°C for 30 s; reaction were carried out for 40 cycles. For data analysis, the relative expression levels of PTK6 were given by 2^-△Ct^,where △Ct = Ct (unknown) - Ct (internal control).

### Western blotting analysis

Equal amounts of whole cell lysates were separated by electrophoresis on a 10.5% SDS polyacrylamide gel electrophoresis (PAGE) and electrotransferred on a polyvinylidene difluoride (PVDF) membrane (Pall, Port Washington, New York, USA). The membrane was blocked with 5% skimmed milk for 1.5 hours (h). Then we incubated the tissues with a primary rabbit polyclonal antibody against human PTK6 (1:500 dilution; Abgent, USA) overnight at 4°C. The proteins bands were visualized using an enhanced chemiluminescent western blot Kit (Amersham, UK). The membranes were stripped and probed with a mouse monoclonal antibody against human GAPDH (1:4000 dilution; Santa Cruz Biotechnology, USA) to confirm equal loading of the samples.

### Immunohistochemistry staining

Formalin-fixed, paraffin-embedded NPC samples were cut into 4-μm thick sequential sections. Then the sections were baked for 3 hours at 58°C. After being deparaffinized in xylenes and rehydrated with graded alcohol to distilled water. We immersed these sections in 3% hydrogen peroxide for 10 min to block endogenous peroxidase activity at room temperature, and then boiled the sections in Citrate Antigen Retrieval Solution (PH=6.0) for 5 min in a high-pressure cooker for antigen retrieval. After the temperature of this retrieval solution return to room temperature, the sections were incubated with diluted rabbit polyclonal anti-PTK6 antibody (1:200 dilution;Abgent, USA) overnight at 4°C in a moist chamber. The next day, after being washed in phosphate buffered saline add Tween-20 (PBST), the sections were incubated with a secondary antibody for 30 min at 37°C and then washed in PBST twice, followed with a 2 min staining in DAB (3,3-diaminobenzidine) for protein detection. The sections were counterstained with Mayer’s hematoxylin to stain nucleus and were finally dehydrated and mounted. A negative control was obtained by replacing the primary antibody with a normal rabbit IgG.

### Evaluation of IHC

The immunoreactivities were scored separately by two pathologists blinded to the clinical parameters. Tumor cell percentage were scored as follows: 0, negative or less than 10% positive tumor cells; 1, 10-25% positive tumor cells; 2, 26-60% positive tumor cells; 3, more than 60% positive tumor cells. Staining intensity was categorized: 0, no staining; 1, weak staining; 2, moderate staining and 3, strong staining. The two individual parameters were multiplied. Then we can get an immunoreactivity score (IRS) ranging from 0 to 9. All results were confirmed by at least 2 pathologists in a double-blind analysis. An optimal cut-off value for high and low expression was selected on the basis of a measure of heterogeneity with the log-rank test statistical analysis with respect to overall survival (OS). For PTK6, the optimal cutoff value was determined: an IRS ≤5.0 defined tumors with low expression, and IRS > 5.0 indicated high expression.

### Statistical analysis

Statistical analysis was performed using SPSS software, version 17.0 (SPSS, Chicago, USA). The correlation between PTK6 expression and clinicopathological status of NPC patients was assessed by chi-square test. Survival curves for both PTK6 high-expression and PTK6 low-expression patients were plotted using the Kaplan-Meier analysis and log-rank test. Univariate and multivariate regression analysis were performed with the Cox proportional hazards regression model to determine the effect of particular prognostic factors on survival. A P-value less than 0.05 was considered as statistically significant in all cases.

### Plasmids and transient transfection

The full-length human PTK6 was cloned into pcDNA3.1 vector with BamHI site and EcoRI site. HNE1 cells (2.5×10^5^) were seeded in 6-well plates at 60% confluence, and then transient transfected with pcDNA3.1 empty vector or pcDNA3.1-PTK6 plasmid using Lipofectamine 2000 (Invitrogen, USA) according to the instructions provided by the manufacturer. After six hours incubation at 37°C with 5% CO_2_, the transfection medium was replaced with 2 ml fresh culture medium. Cells were collected for western blotting, quantitative RT-PCR, proliferation and colony formation assays at 36 hours post-transfection.

### Cell proliferation assay and colony formation assay

MTT (methyl thiazolyl tetrazolium, Sigma-Aldrich, USA) assay was used to determine cell proliferation. For MTT assay, HNE1-pcDNA3.1-vector (control) cells and HNE1- pcDNA3.1-PTK6 (PTK6 overexpression) cells were cultured in 96-well plates at an initial density of 1×10^3^. Then the MTT solution (20 μl, 5 mg/ml in PBS) was added directly into each well and the cells were incubated at 37°C for 4 hours. The medium was removed and 200 μl of DMSO (dimethyl sulfoxide) was added to each well. After 10 minutes of vibration mixing, the optical density (OD) was measured at 570 nm with a microplate reader. The OD value of cell numbers were measured every 24 hours from the first day (day 1) to the fifth day (day 5). The experiment was repeated three times.

For colony formation assay, cells were plated at a density of 200 cells/well in six-well plates, and cultured with RPMI-1640 medium with 5% fetal bovine serum for 9 days. The medium was changed every 3 day for 9 days until most visible colonies had expanded with more than 50 cells. Colonies were simultaneously fixed in methanol for 10 min, and stained with crystal violet for 10 min. After washing out the dye, the colonies were manually counted and the plates were photographed. Three independent experiments were carried out for the assay.

## Results

### The expression of PTK6 in NPC cell lines and fresh biopsies

**Figure 1:**
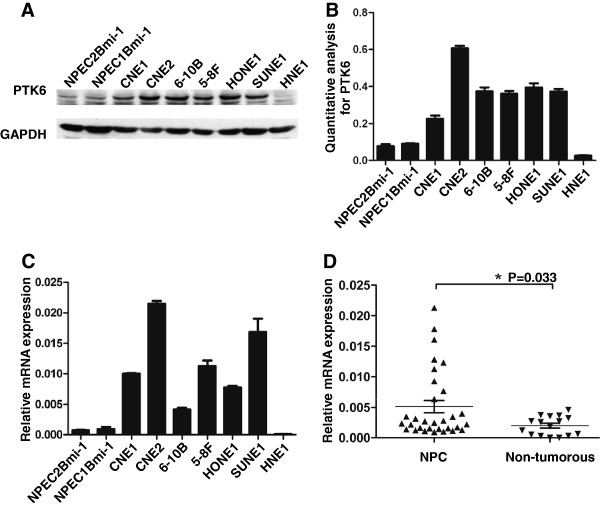
**Expression of PTK6 in cell lines and nasopharyngeal tissues.** (**A**) PTK6 expression was higher in most of NPC cell lines include CNE1, CNE2, 6-10B, 5-8F, HONE1 and SUNE1 than in Bmi-1/NPEC1 and Bmi-1/NPEC2 by Western blot analysis. (**B**) Quantitative analysis of PTK6 protein as mean (±SD) by normalizing to the expression of GAPDH. n=three different experiments. (**C**) Quantitative RT-PCR detection showed that in those 6/7 NPC cell lines presented higher mRNA expression level of PTK6 than that in those two NPECs. n=three independent experiments. (**D**) The mRNA expression levels of PTK6 from 31 NPC patients and 16 non-tumorous tissues were determined by quantitative RT-PCR (P= 0.033).

To examine the expression pattern of PTK6 in NPC, we first performed a western blotting analysis using a monoclonal antibody against PTK6 on 6 NPC cell lines including CNE1, CNE2, 6-10B, 5-8F, HONE1, SUNE1, and HNE1 and two immortalized nasopharyngeal epithelial cells (NPEC1 Bmi-1 and NPEC2 Bmi-1). The expression of PTK6 protein was barely observed in the non-tumorous cells. However, most of the NPC cell lines showed highly expression levels of PTK6 protein except HNE1, a highly differentiated NPC cell line (Figure 1A and B). To evaluate whether the enhanced PTK6 expression was occurred at transcriptional level, we performed a quantitative RT-PCR analysis on the same cell lines. Consistently, most of NPC cell lines determined except HNE1 showed higher expression of PTK6 mRNA than that in non-tumorous cells (Figure 1C). To further examine whether the high expression of PTK6 occurred in NPC patients, we performed quantitative RT-PCR on 31 fresh NPC biopsies and 16 non-tumorous nasopharyngeal epithelial biopsies. As shown in Figure 1C, unlike to the low PTK6 mRNA expression in all of the non-tumorous biopsies, NPC tissues showed a various expression level of PTK6 mRNA. The mean expression level in NPC tissues is significantly higher than the level in non-tumorous tissues (Figure 1D). In conclusion, PTK6 was upregulated in NPC cell lines and also in some NPC tissues.

### PTK6 Expression in NPC and normal nasopharyngeal epithelial tissues by IHC analysis

**Figure 2:**
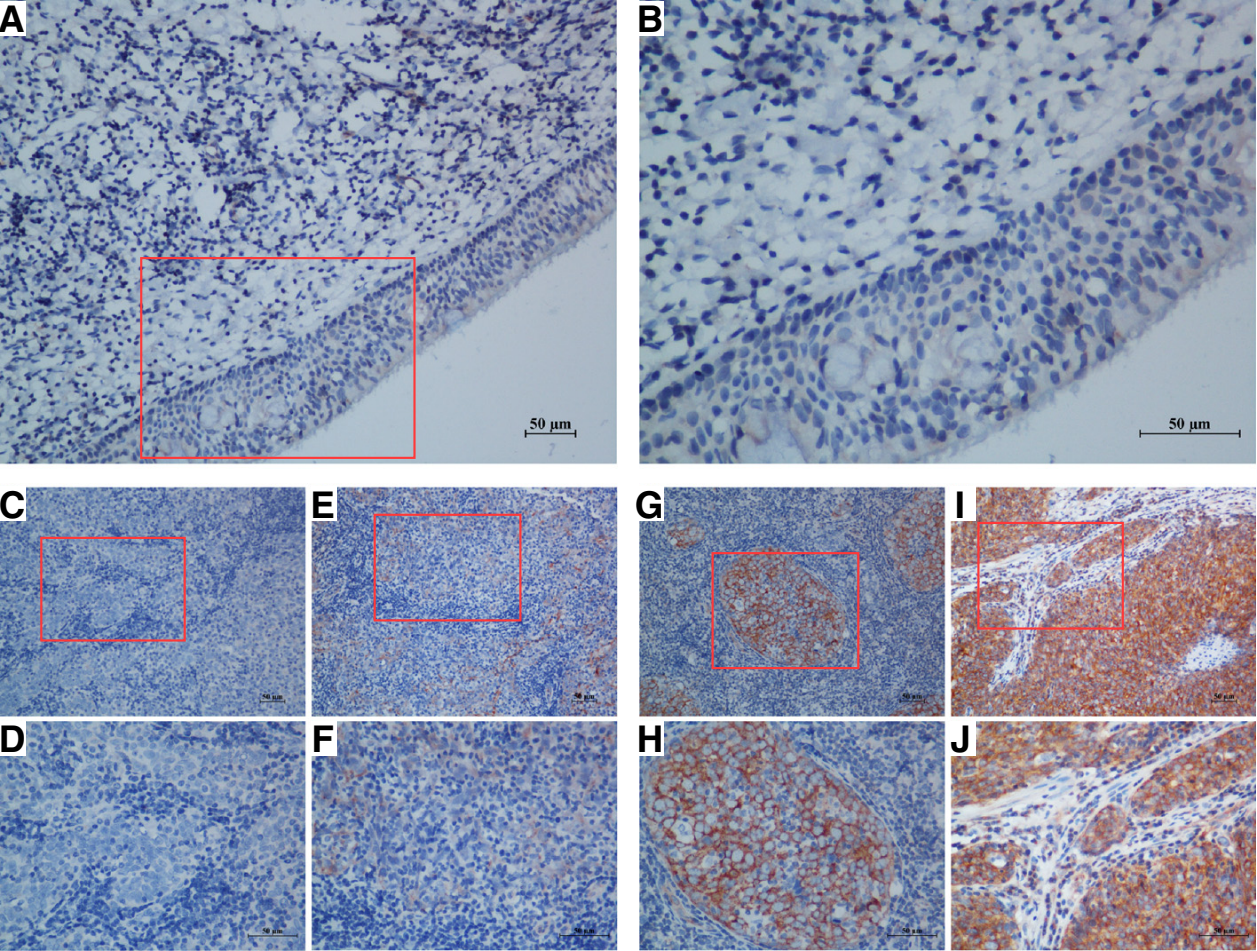
**Expression of PTK6 in NPC tissues by immunohistochemistry staining.** (**A** and **B**) negative PTK6 staining in normal nasopharyngeal epithelium tissue (negative control) (**A**) 200X, (**B**) 400X; (**C** and **D**) negative staining of PTK6 in NPC tissue (**C**) 200X, (**D**) 400X; (**E** and **F**) weak staining of PTK6 in cytoplasm (**E**) 200X, (**F**) 400X; (**G** and **H**) moderate staining of PTK6 in cytoplasm (**G**) 200X, (**H**) 400X; (**I** and **J**) strong staining of PTK6 in cytoplasm (**I**) 200X, (**J**) 400X.

To examine the expression pattern of PTK6 in archive NPC tissues, we performed the IHC analysis with the specific antibody in 178 NPC and 10 normal nasopharyngeal epithelial tissues. The representative results of IHC are shown in Figure 2. In our study, we observed no staining in these 10 cases of normal nasopharyngeal epithelial tissue (Figure 2A and B). However, among the 178 cases of NPC, we observed staining of PTK6 in almost all NPC except one sample (Figure 2C and D). Among them, 27 (15.2%) cases showed weak intensity (Figure 2E and F), 62 (34.8%) cases showed moderate staining (Figure 2G and H), and 88 (49.4%) cases showed strong intensity (Figure 2I and J). The IRS was multiplied by the score of tumor cell percentage and the score of staining intensity as we described before. We also found that PTK6 were largely localized in the cytoplasm of NPC cells.

### Association of PTK6 expression with clinicopathological features and Cox proportional hazards survival analysis

To determine whether the PTK6 expression was correlated with clinical pathological characteristics, all the samples were separated into either low or high PTK6 expression groups, on the basis of their expression levels according to the cutoff score [43]. In our study, the cutoff score for the expression of PTK6 was set at 5.0. As a result, NPC with the score > 5.0 were defined as high expression of PTK6, while the score ≤5.0 were designated as low expression. High expression level of PTK6 was observed in 113/178 (63.5%) of NPC samples. PTK6 expression was significantly associated with tumor size (T classification) (P<0.001, Table 2), clinical stage (P<0.001, Table 2), and metastasis (P=0.016, Table 2) in those all NPC patients; however, there was no significant correlation between PTK6 expression and other clinicopathological features, such as patient age, sex, lymphoid nodal states (N classification), local-regional relapse, and WHO classification (P>0.05, Table 2).

**Table 2:** Correlation between PTK6 expression and clinicopathologic characteristics in NPC cases

**Characteristics**	**PTK6 expression**		**P-value**
	**Low (n = 65) (%)**	**High (n = 113) (%)**	
**Age (years)**			
≤46	39 (60.0)	60 (53.1)	
>46	26 (40.0)	53 (46.9)	0.434
**Sex**			
Female	14 (21.5)	31 (27.4)	
Male	51 (78.5)	82 (72.6)	0.474
**Tumor size**			
T1+T2	33 (50.8)	25 (22.1)	
T3+T4	32 (49.2)	88 (77.9)	<0.001^*^
**Lymphoid nodal states**			
N0-1	34 (52.3)	58 (51.3)	
N2-3	31 (47.7)	55 (48.7)	1.000
**Clinical stage**			
I+II	22 (33.8)	11 (9.7)	
III+IV	43 (66.2)	102 (90.3)	<0.001^*^
**Local-regional relapse**			
Yes	6 (9.2)	17 (15.0)	
No	59 (90.8)	96 (85.0)	0.355
**Metastasis**			
Yes	6 (9.2)	27 (23.9)	
No	59 (90.8)	86 (76.1)	0.016^*^
**WHO histological classification**			
NKUC	62 (95.4)	107 (95.6)	
NKDC	3 (4.6)	6 (4.5)	0.748

^*^Statistically significant difference.

Abbreviations *NPC* nasopharyngeal carcinoma, *PTK6* protein tyrosine kinase 6, *WHO* World Health Organization, *NKU*C non-keratinizing undifferentiated carcinoma, *NKDC* non-keratinizing differentiated carcinoma.

Univariate Cox proportional hazard regression analysis showed that PTK6 expression (P<0.001), age (P=0.003), metastasis (P<0.001), local-regional relapse (P=0.017), lymphoid nodal states (N classification) (P=0.039) and clinical stage (P=0.003) were significantly associated with five-year overall survival (Table 3). The features were also significant in a multivariate Cox proportional hazards regression model (Table 3). After multivariate adjustment for above significant clinicopathological features, PTK6 was an independent and unfavorable factor (HR: 2.038; 95% confidence interval: 1.051-3.951; P=0.035). In addition, age (HR: 1.900; 95% confidence interval: 1.165-3.079; P=0.010), metastasis (HR: 5.290; 95% confidence interval: 3.122-8.962; P<0.001), local-regional relapse (HR: 2.427; 95% confidence interval: 1.303-4.519; P=0.005) and clinical stage (HR: 3.343; 95% confidence interval: 1.057-10.574; P=0.040), respectively, were defined as independent prognostic predictors for five-year overall survival (Table 3).

**Table 3:** Univariate and multivariate Cox regression analysis of different prognostic variables in NPC patients

**Variable**	**Subset**	**HR (95%) CI**	**P-value**
**Univariate analysis (N=178)**			
PTK6 expression	High versus Low	3.519 (1.918-6.458)	<0.001^*^
Age (years)	> 46 versus ≤ 46	2.046 (1.278-3.277)	0.003^*^
Sex	Female versus Male	0.872 (0.512-1.485)	0.614
Metastasis	Yes versus No	4.735 (2.919-7.679)	<0.001^*^
Local-regional relapse	Yes versus No	1.999 (1.129-3.539)	0.017^*^
**Tumor size**	T1+T2 versus T3+T4	1.477 (0.872-2.502)	0.147
Lymphoid Nodal states	N0-1 versus N2-3	1.657 (1.072-2.672)	0.039^*^
Clinical stage	I + II versus III + IV	4.599 (1.676-12.619)	0.003^*^
WHO histological classification	NKDC versus NKUC	1.244 (0.404-3.834)	0.703
**Multivariate analysis (N=178)**			
PTK6 expression	High versus Low	2.038 (1.051-3.951)	0.035^*^
Age (years)	> 46 versus ≤ 46	1.900 (1.165-3.079)	0.010^*^
Metastasis	Yes versus No	5.290 (3.122-8.962)	<0.001^*^
Local-regional relapse	Yes versus No	2.427 (1.303-4.519)	0.005^*^
Lymphoid Nodal states	N0-1 versus N2-3	1.372 (0.823-2.288)	0.225
Clinical stage	I + II versus III + IV	3.343 (1.057-10.574)	0.040^*^

^*^Statistically significant difference

Abbreviations *PTK6* protein tyrosine kinase 6, *NPC* nasopharyngeal carcinoma, *HR* hazard ratio, *CI* confidence interval.

### Correlation of PTK6 expression and overall survival

**Figure 3:**
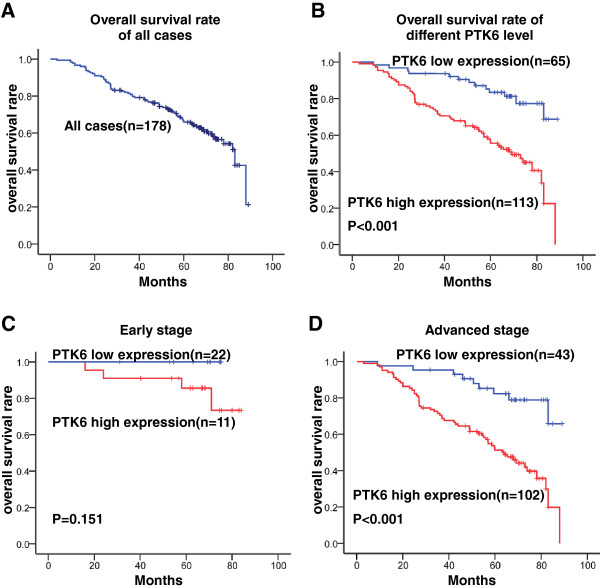
**Kaplan-Meier survival curve and log-rank test analysis showing the association between PTK6 expression and NPC patient survival.** (**A**) The five-year overall survival (OS) rate was 53.9% of 178 NPC patient; (**B**) High PTK6 expression level was significantly correlated to OS (P<0.001) in all NPC patients. (**C**) Cases were stratified by clinical stage. No significant difference in five-year OS rate was found between PTK6 high-expression and low-expression in NPC patients at early stages (StageI+II); (**D**) High PTK6 expression level was significantly associated with OS (P<0.001) in NPC patients at late stages (Stage III+IV).

The five-year OS rate of the cohort of 178 NPC patients was 53.9% (Figure 3A). The prognostic value of PTK6 was evaluated through estimation of OS using Kaplan-Meier and log-rank test analyses. The result showed that high PTK6 expression was significant related to poor OS compared to low PTK6 expression (47.8% versus 80.0%, P<0.001, Figure 3B). Next, we stratified the clinical stages into two groups that include early stages (I + II) and advanced stages (III + IV), and found that the association between high PTK6 expression and shorter OS were significantly stronger in patients at advanced stages than in early stages. In the advanced stages, the 5-year OS rate of late stages patients with high or low expression of PTK6 was 42.2% or 79.1%, respectively (P<0.001, Figure 3C). However, in the earlier stages, the 5-year OS rate was 100% and 81.8% among high expression and low expression patients (P=0.151, Figure 3D).

### Overexpression of PTK6 in NPC cell line HNE1

**Figure 4:**
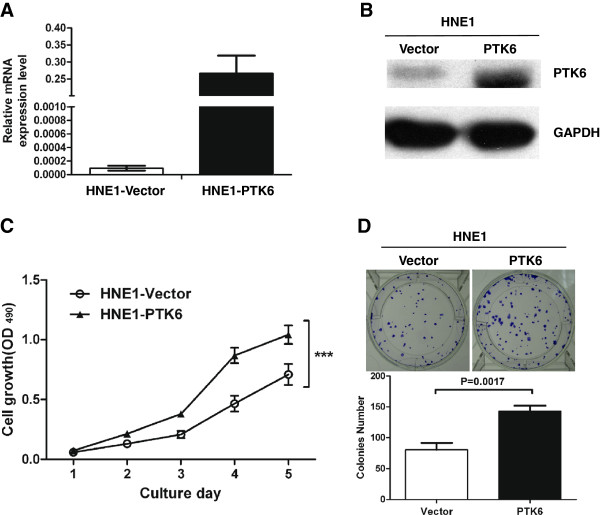
**PTK6 overexpression simulates the proliferation and transforming ability of NPC cells.** (**A**) PTK6 overexpression in HNE1 cells at 36 hours post-transfection. HNE1 cells were transfected with pcDNA3.1-PTK6 and the control plasmid pcDNA3.1-empty vector, respectively. A dramatically increased mRNA level of PTK6 was detected in pcDNA3.1-PTK6 transfected cells by quantitative RT-PCR. (**B**) Western blotting analysis showed that the protein level of PTK6 in HNE1 cells after transfection. (**C**) The growth curves of HNE1 cell line. After transient transfection of PTK6, the PTK6-overexpression cells grew significantly faster than control cells from day 2 to day 5 (P<0.001, marked by *). Error bar =SD. (**D**) Representative pictures of colony formation assay of the HNE1 cell line transfected with pcDNA3.1-PTK6 and the control plasmid pcDNA3.1-empty vector. Upon the transient transfection expression of PTK6, HNE1 cells formed more colonies compared to the control cells. Error bar =SD.

HNE1 NPC cells containing relatively lower endogenous PTK6 levels were transfected with pcDNA3.1-PTK6 to achieve overexpression of PTK6. At 36 hours post-transfection, both mRNA level and protein level of PTK6 were higher in PTK6-overexpression cells compared to the control cells transfected with empty vector by quantitative RT-PCR and western blot analysis, respectively (Figure 4A and 4B).

### PTK6 Enhances the proliferation and colony formation ability of NPC cells

To investigate the effect of PTK6 on cellular proliferation in HNE1 cells, the MTT assay was performed after overexpressing PTK6. As shown in Figure 4C, the growth curves from the day 1 to 5 demonstrated that HNE1 cells with overexpression of PTK6 grew faster than control cells, suggesting that overexpression of PTK6 can enhance the proliferation of the NPC cells.

Then we explored the colony formation ability of PTK6-overexpressed HNE1 by the colony formation assay, and found that PTK6-overexpression cells formed more colonies than those of the control cells (Figure D, upper panel). There is a significant differences in the number of colonies between the vector control cells and the PTK6-expression cells (P=0.0017; Figure D, lower panel).

## Discussion

NPC is a malignant tumor that originates from the upper lining and lateral wall of nasopharynx. Approximately 70% of NPC patients often diagnosed at late stages when the time of diagnosis, leading to a high rate of local-regional relapse and metastasis after radiotherapy alone [44,45]. NPC high death rate is mainly due to tumor metastasis despite the new treatment that combining radiotherapy with chemotherapy [46]. Therefore, novel molecular biomarkers for confirming tumor metastasis and predicting prognosis to improve the cure rate are eagerly needed.

In our study, we observed a high expression of PTK6 in most NPC cell lines include CNE1, CNE2, 6-10B, 5-8F, HONE1 and SUNE1 at both mRNA and protein level, but not in a highly differentiated NPC cell line HNE1, NPEC1 Bmi-1 or NPEC2 Bmi-1 cells. The mRNA level of PTK6 expression are higher in NPC biopsy samples than in non-tumorous nasopharyngeal mucosa biopsies. Moreover, the IHC result showed that PTK6 was not expressed in normal nasopharyngeal epithelium, but presented an increased expression in most NPC samples. Taken together, it suggested that PKT6 might play an important role in NPC.

Furthermore, the high expression of PTK6 was significantly correlated to tumor size (T classification), clinical stage, and metastasis, respectively (P<0.05, Table 2). We also showed that high expression of PTK6 was associated with poor 5-year overall survival rate of NPC (P<0.001, Figure 3B). Moreover, compared to the early stages, a strong correlation between high PTK6 expression and short survivals was found at advanced stages (P<0.001, Figure 3A and D), indicating that PTK6 might involve in progression and metastasis of NPC. These findings are in accordance with some previous reports, such as breast cancer [47,48], non-small cell lung cancer [33], ovarian cancer [31], colon cancer [20], head and neck cancers [32] and metastatic melanoma cells [34].

Since tumor metastasis is the major cause of advanced NPC, it is urgent to identify a biomarker for diagnosis of NPC and find a potential therapy target for NPC metastasis. In the current study, we showed that a high expression of PTK6 in most of NPC cell lines and NPC tissue samples, suggested that PTK6 may serve as a biomarker for advanced stages patients and as a possible therapy factor. Multivariate Cox proportional hazards survival analysis indicated that high PTK6 expression in NPC tumor tissues was an independent and unfavorable factor for poor prognosis of NPC patients (P=0.035, Table 3). We also confirmed that advanced increased age (P=0.010), metastasis (P<0.001), local-regional relapse (P=0.005), and advanced clinical stage (P=0.004) were the independent predictive factors for survival, these result were consistent with previous studies [49-51]. All the results suggested that PTK6 could be a useful biomarker for the prognosis and metastasis of NPC.

PTK6 has a structural homology with c-Src-family tyrosine kinase and consists of a tyrosine kinase domain that is subject to autophosphorylation and autoinhibition, as well as SH2 and SH3 domains that are implicated in protein interactions and autoregulation. However, distinct from c-Src, PTK6 lacks an N-terminal SH4 domain required for fatty acid acylation and membrane localization, thereby rendering that PTK6 could locate in both cytoplasmic and nuclear compartments. Soluble PTK6 seems to play an important role in mediating signaling pathways and many aspects of cell biology across different cellular contexts. Taken together, these suggest that the functions of PTK6 depend on not only cell types, but also its intracellular localization. The location of PTK6 is variable in different cells. Schmandt RE et al. used IHC to demonstrate that PTK6 was high expressed in 70% of the ovarian cancer, but was absent in the surface epithelia of normal ovarian. The also found that PTK6 primarily located at the cytoplasm of ovarian cancer cells, however, in a subset of tumor cells, staining was observed in the nuclei [31]. Fan, C et al. also found the similar results, they found the expression of PTK6 was significantly higher in cytoplasm of NSCLC compared to in the nuclei. Furthermore, the cytoplasmic expression of PTK6 in normal lung tissue cells was lower than that in NSCLC [33]. Interesting, it is reported that PTK6 is located in the nuclei of normal prostate epithelium and well-differentiated prostate tumor, but it is located in the cytosol of poorly differentiated prostate carcinoma [19]. Recently, Ma et al. identified PTK6 mRNA expression was significantly reduced in esophageal squamous cell carcinoma (ESCC) due to epigenetic modification and work as a tumor suppressor via in vivo and in vitro studies. Unlike in the cases for ovarian cancer, NSCLC and prostate carcinoma, the found PTK6 located in both cytoplasm and nuclei fractions in ESCC [18]. In our study, PTK6 was found to be located primarily in cytoplasmic compared to in the nuclei fractions in NPC, this result was similar with the previous study of the expression of PTK6 in ovarian carcinoma, NSCLC and poorly differentiated prostate cacinoma [19,31,33]. It has been suggested that nuclear PTK6 is possibly related to growth regulation in normal epithelium; however, cytoplasmic PTK6 may activate oncogenic signaling pathways [19,52]. So, a large scale of clinical investigation and further experimental study are still necessary to elucidate the exact role of PTK6 in NPC.

It is reported that PTK6 has been demonstrated to link with cellular differentiation, apoptosis, migration and wound healing in normal epithelium. However, PTK6 also participates in cancer progression by sensitizing cells to mitogenic signals, enhancing proliferation, migration/invasion, and anchorage-independent survival in tumor tissues [53]. Interesting, according to several in vitro studies using both knockdown and overexpression systems, PTK6 was shown to increase proliferation, anchorage-independent growth, cell migration, and tumor growth in breast cancer models. Nevertheless, it has been suggested that a significant correlation between PTK6 and the estrogen receptor as well as overexpression of PTK6 corresponds to better prognosis [54,55], these provides evidence for that PTK6 may associate with the cell type and degree of differentiation of cancer cells in breast tumors. It is appeared that different expression patterns of ligands, receptors, as well as PTK6 substrates and binding partners affect the specificity of PTK6 action. In our study, the results of MTT assay and colony formation assay showed that overexpression of PTK6 can enhance cell proliferation and colony formation ability and high expression of PTK6 cause poor prognosis in NPC. However, the mechanism of the function of PTK6 in NPC is still unknown. Further studies are needed to characterize the apparent context- and tissue-specific functions of PTK6 in both normal tissues as well as tumors and clarify the contributions of PTK6 to initiation and progression of epithelial cancers.

Shen et al. showed that PTK6 induced tumor proliferation, invasion and migration by triggering EGF-mediated phosphorylation of p190RhoGAP, leading to Ras activation and RhoA inactivation in MDA-MB231 breast cancer cells [56]. Moreover, it has been reported that high expression level of EGFR was significantly associated with T stages, clinical stages, local recurrence, and poor prognosis of the NPC patients [12,57,58]. Furthermore, PTK6-induced paxillin phosporylation promoted Rac activation via CrkII and cellular migration and invasion [59]. Additionally, kinesin-2 subunit KAP3A was necessary for cell migration promoted by PTK6 [60]. Carol et al. demonstrated that PTK6 mediated HGF/MET-induced cell migration in breast cancer [48,53,61]. Interestingly, it is well known that overexpression of Met was a poor prognosis biomarker of NPC, correlated with lymph node metastasis, and its aberrant activation depends on paracrine HGF from stoma cells rather than an autocrine loop or activating mutation [62-64]. Based on our results, PTK6 was significantly associated with T classification, clinical stage and metastasis in NPC. In addition, overexpression of PTK6 can stimulate cell proliferation and colony formation ability, suggesting that PTK6 might play an important role in NPC progression and metastasis, probably through sustaining and activating EGF pathway or through the activation of downstream pathways of HGF/MET in NPC. Therefore, we will deeply investigate the mechanisms underlying PTK6-mediated progression and metastasis of NPC in the future study, by identifying the receptor, adapters and target proteins and pathways of PTK6.

## Conclusion

In summary, we observed the highly expressed PTK6 in NPC and overexpression of PTK6 contributed increases in cell proliferation and colony formation ability of NPC cells, and demonstrated that high level of PTK6 is closely correlated with poor prognosis of NPC, suggesting that PTK6 may serve as a useful biomarker to determine the prognosis of NPC and provide a potential therapeutical target for NPC treatment.

## Abbreviations

PTK6: Protein tyrosine kinase 6; NPC: Nasopharyngeal carcinoma; IHC: Immunohistochemistry staining; UICC: International Union Against Cancer; AJCC: American Joint Committee on Cancer; NPECs: Immortalized nasopharyngeal epithelial cell lines; GAPDH: Glyceraldehyde-3-phosphate dehydrogenase; PBST: Phosphate buffered saline add Tween-20; IRS: Immunoreactivity score; OS: Overall survival; WHO: World Health Organization; NKUC: Non-keratinizing undifferentiated carcinoma; NKDC: Non-keratinizing differentiated carcinoma; HR: Hazard ratio; CI: Confidence interval.

## Competing interests

The authors declare that they have no conflict of interest.

## Authors’ contributions

LNL designed and performed the experiments of IHC, quantitative RT-PCR, western blot assays, carried out celluar studies, analyzed the results and draft the manuscript. PYH collected the biopsies specimens, analyzed the results and participated in drafting of the manuscript. ZRL carried out a part of celluar studies. LJH participated collected the biopsies specimens. JZL collected the paraffin-embedded specimens and the Characteristics of the patients and help to guide the experiment of IHC. MZL and LQT participated in a part of celluar studies and the data collection. BHZ and QZ conceived the study, participated in its design and coordination and helped to draft the manuscript. MSZ guided the editing of the manuscript. All authors read and approved the final manuscript.
